# Integrated physiological, transcriptomics and metabolomics analysis revealed the molecular mechanism of *Bupleurum chinense* seedlings to drought stress

**DOI:** 10.1371/journal.pone.0304503

**Published:** 2024-06-06

**Authors:** Xiaohan Feng, Yan Sun, Ya Fan, Quanfang Zhang, Xun Bu, Demin Gao

**Affiliations:** 1 School of Pharmacy, Shandong University of Traditional Chinese Medicine (TCM), Jinan, China; 2 Institute of Crop Germplasm Resources, Shandong Academy of Agricultural Sciences, Jinan, China; Huazhong University of Science and Technology, CHINA

## Abstract

Drought stress is a prominent abiotic factor that adversely influences the growth and development of *Bupleurum chinense* during its seedling stage, negatively impacting biomass and secondary metabolite production, thus affecting yield and quality. To investigate the molecular mechanism underlying the response of *B*. *chinense* seedlings under drought stress, this study employed comprehensive physiological, transcriptomic, and metabolomic analyses. The results revealed that under drought stress, the root soluble sugar and free proline content in *B*. *chinense* seedlings significantly increased, while the activities of SOD, POD, and CAT increased in the leaves. These findings indicate the presence of distinct response mechanisms in *B*. *chinense* to cope with drought stress. Integrated analysis further identified significant correlations between genes and metabolites related to amino acid biosynthesis in the leaves, as well as genes and metabolites associated with acetaldehyde and dicarboxylic acid metabolism. In the roots, genes and metabolites related to plant hormone signaling and the tricarboxylic acid (TCA) cycle showed significant correlations. These findings provide vital views into the molecular-level response mechanisms of *B*. *chinense* under drought stress. Moreover, this study establishes the groundwork for identifying drought-tolerant genes and breeding drought-resistant varieties, which could improve the drought tolerance of medicinal plants and have broader implications for agriculture and crop production in water-scarce areas.

## Introduction

Contemporary global warming presents grave challenges to ecosystems, agriculture, and water resources worldwide [1]. A critical aspect of this environmental upheaval is drought, which, as a byproduct of climate change, significantly impacts the growth and distribution of medicinal plants [2]. Predictions indicate that future droughts in China, especially in the main producing areas of Chinese medicinal materials, will become increasingly frequent, prolonged, and severe [3,4]. This trend poses a serious challenge to the cultivation and development of medicinal herbs, highlighting the urgent need to enhance our understanding of how these plants respond to these environmental stresses.

Drought stress is known to induce oxidative damage and lipid peroxidation in plant membranes, leading to metabolic disruptions. The typical responses to drought include stunted plant growth, decreased turgor, and reduced transpiration rates [5,6]. Plants adapt by producing osmotic substances like proline and soluble sugars for osmoregulation, cell turgor maintenance, and metabolic stabilization [7]. Moreover, the accumulation of reactive oxygen species (ROS) can activate antioxidant enzymes (POD, SOD, CAT), which mitigate oxidative damage under drought stress [8,9]. Drought also triggers specific signaling and metabolic pathways, such as protease activation and flavonoid production [10,11], with the ABA signaling pathway playing a crucial role in stomatal closure and stress resistance gene expression in plants [12,13]. For instance, exogenous ABA inhibits the expression of genes involved in saponin biosynthesis to alleviate drought-induced stress of *Bupleurum chinense* [14].

*B*. *chinense*, a perennial herb commonly grown in the dry and semi-arid regions of China, is significantly affected by its cultivation environment [15–17]. Although existing research indicates that drought stress can trigger the saponins in roots and biosynthesis of flavonoids in leaves [18–20], this stress presents considerable challenges to the early stages of breeding and seedling cultivation of *B*. *chinense* [21,22]. Such conditions adversely impact on subsequent growth, secondary metabolite production, and medicinal quality of the plant [20,23,24]. *B*. *chinense* seedlings are particularly susceptible to drought stress, a critical phase impacting their development and growth [25]. Therefore, there is a pressing need to concentrate research efforts on the seedling stage to thoroughly understand the intricate interactions between drought tolerance and the physiological, biochemical, genetic, and metabolic responses of *B*. *chinense*.

In light of these considerations, this study provides a comprehensive analysis of the physiological, transcriptomic, and metabolomic responses of *B*. *chinense* seedlings to drought stress. Through this integrative approach, we aim to identify key metabolites and genes related to drought tolerance, thereby unraveling the intricate molecular mechanisms underlying the plant’s response. This research is pivotal in developing strategies for the sustainable cultivation and breeding of drought-resistant Bupleurum varieties, ensuring the continued production and quality of this important medicinal plant.

## Materials and methods

### Plant materials and stress treatment

*B*. *chinense* seeds were harvested from Changzhi, Shanxi Province (E 112°56′8″, N 35°56′47″) in August 2020. Subsequently, they were cultivated in the botanical garden of Shandong University of TCM (E116°35′07″, N 36°33′20.98″). The identification of *B*. *chinense* was carried out by Dr. Lingchuan Xu, an expert in medicinal botany at Shandong University of TCM in China. Voucher specimens and related information were deposited at the Herbarium of Shandong University of TCM, China (STCM2020100809). All materials of *B*. *chinense* used in this study were provided by the institute, and no specific permission was required to collect these samples for research purposes in accordance with the institutional, national, and international guidelines.

The soil moisture during the experiment was maintained at approximately 20.00%, with a soil pH of 7.22. From August 2021 to April 2022, the average daytime temperature, average night temperature, maximum temperature and minimum temperature were 16.56°C, 7.88°C, 35.00°C and -19.00°C, respectively. In the second year, when the seedlings reached a height of approximately 6–7 cm, they were transplanted into a basin with dimensions of 20 cm in both diameter and height. The seedlings were watered daily to maintain a water content in the basin is 16.13 ± 3.38% (equivalent to 80% of the field capacity). After 20 consecutive days of cultivation, the experimental group was subjected to continuous drying and was not watered, while the control group was watered normally. On the 10^th^ day, the leaves started to curl as the soil moisture content reached 7.03 ± 1.24%. At this stage, the seedlings were delicately extracted from the soil to maintain root integrity. Subsequently, they were promptly rinsed with ultra-pure water and positioned on autoclaved filter paper. Each leaf and root of *B*. *chinense* was then divided into two parts. One part was rapidly frozen in liquid nitrogen and stored in a refrigerator at -80°C for RNA extraction and metabolite analysis, while the other part was used for physiological and biochemical analysis. For the transcriptome and metabolomics analysis, three and six biological replicas were used, respectively. Details of the collected samples were given in S1 Table.

### Determination of osmolytes

Soluble sugar (SS) was quantified using Plant soluble sugar content test kit (A145-1-1) produced by Nanjing Jiancheng Biology Engineering Institute (Nanjing, China). Fresh roots or leaves of *B*. *chinense* seedlings were prepared following the instructions provided with the kit, and the quantification was carried out at 630 nm using the anthraquinone colorimetric method. Soluble protein contents were quantified using Plant Protein S (PROS) ELISA Kit (MBS9370755) produced by MyBioSource (California, USA) with reference to instructions.

The determination of free proline was determined using the acid ninhydrin colorimetric method [26]. The 200 mg fresh root (or leaf) sample was treated with 10 mL of 3% salicylsulfonic acid solution and heated in a 100°C water bath for 20 min. After cooling, the sample was filtered, and 2 mL of the filtrate was extracted. Subsequently, 2 mL of glacial acetic acid and 3 mL of acid ninhydrin solution were added to the extracted sample, which was then heated in a 100°C water bath for 30 min. After cooling, 5 mL of toluene was added, followed by agitation for 30 s. The red toluene layer was transferred to a colorimetric dish, and its absorbance was measured at 520 nm. The concentration of proline was determined using a standard curve.

### Determination of antioxidant enzyme activity

The 200 mg fresh root (or leaf) sample was crushed in a mortar and pestle using a phosphate buffer and homogenized in an ice bath. Subsequently, the mixture was centrifuged at 4000 rpm for 15 min, and the resultant supernatant was stored in a centrifuge tube at 4°C for subsequent analysis of antioxidant enzyme activity.

The activity of Superoxide Dismutase (SOD) was quantified using the nitroblue tetrazolium (NBT) reaction, with the absorbance recorded at 560 nm [27]. One unit of SOD activity is defined as the amount of enzyme that causes a 50% inhibition of the reduction of p-nitroblue tetrazolium under the experimental conditions. Peroxidase (POD) activity was assessed by measuring the oxidation of guaiacol at an absorbance of 470 nm [28]. For this enzyme, one unit of enzyme activity corresponds to an absorbance change of 0.01 per minute. Catalase (CAT) activity was determined by measuring the UV absorption at 240 nm [29], where one unit of enzyme activity is represented by a change of 0.01 Optical Density (OD) per minute.

### RNA extraction and library construction

Total RNA was extracted from leaves and roots of *B*. *chinense* using Trizol® Reagent (Invitrogen, San Diego, USA). The concentration and purity of the extracted RNA was measured with a Nanodrop 2000 (Thermo Scientific, Massachusetts, USA), and their integrity was confirmed by agarose gel electrophoresis. The cDNA libraries were constructed using Illumina’ s Truseq TM RNA sample prep kit (Illumina, San Diego, USA).

The original sequences obtained by Illumina-Hiseq 2500 were processed by removing low-quality reads that contained more than 5% unknown bases [30]. After quality control, the high-quality sequences were assembled using Trinity (http://trinityrnaseq.sourceforge.net/, Version number: trinityrnaseq-r2013-02-25) to obtain transcripts. The longest sequence from each gene cluster was identified as a unigene for subsequent analysis.

### Transcriptomics data processing

Gene expression was quantified using FPKM, and the identification of differentially expressed genes (DEGs) was based on the criteria of |log2 Fold Change (FC)| ≥ 1.00 and false discovery rate (FDR) ≤ 0.05 [31,32]. The DEGs were functionally annotated using Blast2GO for GO functional classification, and their specific metabolic pathways specific to them were analyzed using the Kyoto Encyclopedia of Genes and Genomes (KEGG) database [33,34]. A threshold of *p* < 0.05 is applied for enrichment analysis of the KEGG pathways. The COG method was employed to annotate the alignment sequences, removing those with e_values greater than 1e^-5^, and selecting the sequences with the best alignment results [35].

### Validation of DEGs by a qPCR analysis

To validate the transcriptome data, real-time fluorescence quantitative PCR (qRT-PCR) were performed on seven DEGs with a| log_2_ratio| between 2 and 10, and high expression levels in the leaves and roots of *B*. *chinense* under drought stress [36]. Primer Premier 5.0 was used to design specific primers. The reaction mixtures consisted of 10 μL of 2 × T5 Fast qPCR Mix (SYBR Green I), 0.8 μL each of 10 μM forward and reverse primers, and 1 μL of cDNA, with the volume supplemented with ddH_2_O to reach a total of 20 μL. The qPCR procedure involved initial denaturation at 95°C for 1 min, followed by 40 cycles of denaturation at 95°C for 15 seconds, annealing at 60°C for 15 seconds, and extension at 72°C for 30 seconds. Each sample was repeated three times. The qPCR procedure included an initial denaturation at 95°C for 1 min, followed by 40 cycles of denaturation at 95°C for 15 seconds, annealing at 60°C for 15 seconds, and extension at 72°C for 30 seconds. Each sample underwent three repetitions. The Actin gene of *B*. *chinense* served as the internal control, and the relative changes in gene expression were assessed using the 2^-ΔΔCT^ method [37].

### Metabolic profiling

One hundred milligrams of sample were extracted with 1.0 mL of water: acetonitrile: isopropanol (1:1:1, v/v). The mixture was vortexed for 60 seconds and then ultrasonically treated at 4°C for 30 min. Afterward, the sample was centrifuged for 10 min at 12000 rpm. The resulting supernatant was placed at -20°C for 1 h, followed by centrifugation at 12000 rpm for 10 min at 4°C. The supernatant was then vacuum-dried and redissolved with 200 μL of 50% acetonitrile. It was subsequently centrifuged at 4°C for 15 min at 14000 rpm [38]. The final supernatant was analyzed using ultra-high-performance liquid chromatography tandem mass spectrometry (UHPLC-MS/MS) with a Thermo-Fisher Exactive mass spectrometer, which consists of an ElectroSpray Ionization source (ESI) and an Orbitrap mass analyzer. The chromatographic column was a Waters HSS T3 (100×2.1 mm, 1.8 μm). The mobile phases utilized in this study comprised 0.1% formic acid (A) and acetonitrile containing 0.1% formic acid (B), with a consistent flow rate of 0.3 mL/min. The column temperature was maintained at 40°C, and a 2 μL injection volume was employed. The elution gradient followed a specified pattern: 0.0–1.0 min A/B (100:0, v/v), 1.0–9.0 min A/B (5:95, v/v), 9–13 min A/B (5:95, v/v), and 13.1–17.0 min A/B (100:0, v/v). ESI source parameters were set as follows: spray voltage at -2.8 kV/3.0 kV, sheath gas pressure at 40 arb, auxiliary gas pressure at 10 arb, sweep gas pressure at 0 arb, capillary temperature set to 320°C, and auxiliary gas heater temperature maintained at 350°C. The samples were maintained at 4°C in an autosampler throughout the analysis [39].

The raw data were processed using Progenesis QI (Waters Corporation, Milford, USA) to generate data matrices for retention time, mass-to-charge ratio, and peak intensity. The main databases used for analysis included http://www.hmdb.ca/, https://metlin.scripps.edu/, and other public and self-built databases. The data preprocessing steps were as follows: 1) Only the variables with more than 80% non-zero values in the two groups of samples were retained; 2) The overall peaks underwent normalization, and variables exhibiting a relative standard deviation (RSD) of ≥ 30% in the quality control (QC) samples were excluded.; 3) The data were log10 transformed to obtain the final data matrix.

### Metabolomics data processing analysis

Multivariate data were analyzed using R (version R-3.4.2), including PCA and OPLS-DA. The robustness of the model was assessed through response permutation testing. The contribution of each variable in the OPLS-DA model was determined by calculating its variable importance in the projection (VIP) value. Metabolites with VIP values greater than 1 were subjected to t-tests at the univariate level to measure the significance, with a *p-*value of less than 0.05 considered statistically significant. The annotation of metabolites involved their identification through the utilization of the KEGG Compound database (http://www.kegg.jp/kegg/compound/). Subsequently, these annotated metabolites were mapped to the KEGG pathway database (http://www.kegg.jp/kegg/pathway.html).

### Correlation analysis of transcriptome and metabolome data

The transcriptome and metabolome were compared to conduct pathway enrichment analysis, and the DEGs and differential accumulation metabolites (DAMs) in each pathway were calculated. Significantly different genes and metabolites were subjected to correlation hierarchical clustering using the R (version R-3.4.2) Heatmap package. The network interactions were established using correlation coefficients, and the data were visualized using Cytoscape v3.9.1.

## Result

### Osmotic regulatory substances and antioxidative enzymes activities

The results showed that the content of soluble sugar and free proline in roots of *B*. *chinense* seedlings significantly increased compared to the control group under drought treatment, while the soluble protein content significantly decreased (Table 1). However, there was no significant difference in soluble sugar content in leaves. These findings indicate that to enhance drought resistance, *B*. *chinense* seedlings can maintain osmotic regulation by adjusting the levels of free proline, soluble sugars, and soluble proteins in both roots and leaves.

**Table 1:** Determination of osmotic adjustment substances in the fresh *Bupleurum chinense* seedlings under drought stress.

Group	Soluble sugar content (mg/g)		Soluble protein content (mg/g)	Free proline content (μg/g)
BL	9.70 ± 0.13^a^	10.20 ± 0.08^a^		31.88 ± 0.05^a^
BDL	9.66 ± 0.13^c^	7.54 ± 0.09^c^		37.98 ± 0.03^c^
BR	6.72 ± 0.07^b^	4.65 ± 0.06^b^		34.79 ± 0.03^b^
BDR	7.61 ± 0.13^d^	3.73 ± 0.03^d^		58.30 ± 0.04^d^

Note: BL, BR, BDL and BDR refer to leaf, root, drought leaf, drought root of *B*. *chinense* respectively. Values are expressed as mean±SD (n = 3). Different lowercase letters indicate significant differences between different samples compared to the control (*p<*0.01).

The activities of antioxidant enzymes (POD, SOD, and CAT) were quantified in response to drought stress (Table 2). The results revealed a significant increase in the activities of SOD, POD, and CAT in the leaves of *B*. *chinense* seedlings after drought stress, while a notable decrease was observed in the roots activities. Specifically, CAT activity increased the most in leaves activity, followed by SOD and POD, indicating that CAT was the main antioxidant enzyme in leaves responding to drought stress. In roots, SOD activity showed the least decrease, followed by CAT and POD. These results suggest that SOD plays a crucial role as an antioxidant enzyme in roots under drought stress.

**Table 2:** Determination of antioxidative enzyme activities in the fresh *Bupleurum chinense* seedlings under drought stress.

Group	SOD (U/g)	POD (U/g)	CAT (U/g)
BL	18.34 ± 0.05^a^	553.57 ± 11.74^a^	393.81 ± 7.14 ^a^
BDL	34.68 ± 0.04^b^	736.19 ± 9.06^b^	1010.48 ± 2.38^b^
BR	17.78 ± 0.08^a^	478.17 ± 4.61^a^	1045.24 ± 16.19^b^
BDR	8.91 ± 0.07^c^	443.73 ± 5.48^a^	810.36 ± 7.02^c^

Note: BL, BR, BDL and BDR refer to leaf, root, drought leaf, drought root of *B*. *chinense* respectively. Values are expressed as mean±SD (n = 3). One unit of enzyme activity is defined as the amount of enzyme that causes a change in absorbance of 0.01 per minute. Different lowercase letters indicate significant differences between different samples compared to the control (*p<*0.01).

### Transcriptome analysis

**Fig 1:**
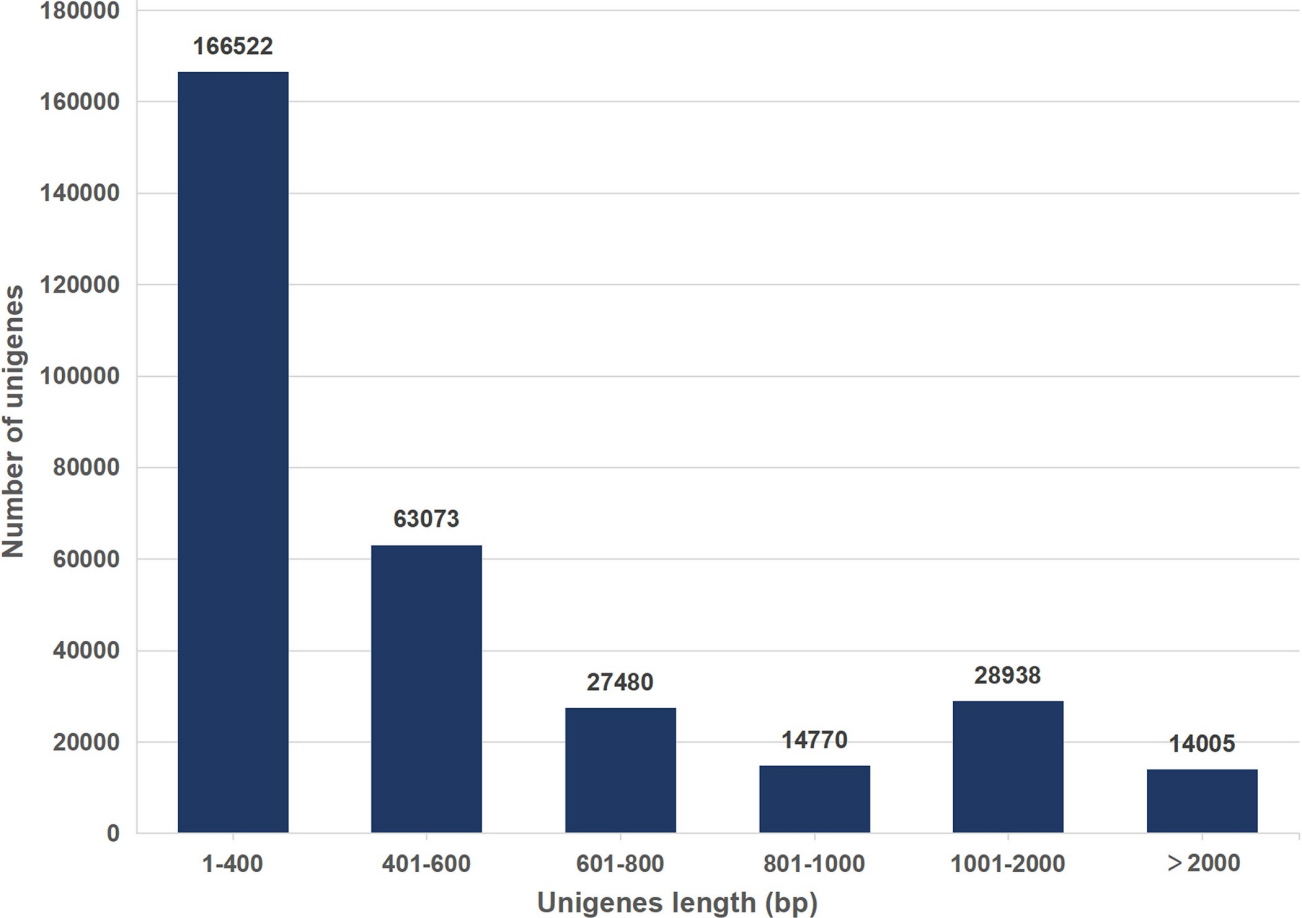
Size distribution of the unigenes in *Bupleurum chinense*.

The RNA extracted from 12 samples of *B*. *chinense* exhibited high quality, as indicated by OD_260/280_ values above 2.0 (S2 Table). In this experiment, a total of 59.82 Gb of clean data was obtained, with an average of 4.98 Gb of clean data per sample (Table 3). The clean data had a Q30 base percentage greater than 5.48% and the GC base content ranged from 44.04% to 45.55%. Using Trinity, the clean data for all samples were de novo assembled, resulting in the assembly of 314788 unigenes, with an average N50 length of 789 bp. Among the assembled unigenes, 229,595 (72.94%) ranged in length from 1 to 600 bp, while 71,188 (22.61%) ranged in length ranged from 601 to 2000 bp (Fig 1). PCA cluster analysis, based on correlation analysis, showed good biological replicates and sample relationships (S1 Fig). Therefore, the transcriptome sequencing data is suitable for further analysis.

**Table 3:** Summary of RNA-Seq data of *Bupleurum chinense*.

Samples	Raw reads number	Clean reads number	Clean bases (bp)	Q20 percentage (%)	Q30 percentage (%)
BL-1a	35,234,272	34,545,236 (98.04%)	5,037,919,736	98.85	95.51
BL-1b	39,567,542	38,911,926 (98.34%)	5,650,054,266	98.95	95.80
BL-1c	44,472,456	43,680,856 (98.22%)	6,364,577,275	98.92	95.72
BR-1a	37,035,734	36,399,496 (98.28%)	5,313,535,634	98.89	95.61
BR-1b	37,419,172	36,733,800 (98.17%)	5,321,935,283	98.90	95.67
BR-1c	36,962,800	36,285,118 (98.17%)	5,244,354,463	98.84	95.48
BDL-1a	26,185,802	25,734,308 (98.28%)	3,751,987,716	98.87	95.57
BDL-1b	36,947,406	36,338,764 (98.35%)	5,302,486,665	98.96	95.85
BDL-1c	37,896,440	37,165,906 (98.07%)	5,388,140,064	98.88	95.59
BDR-1a	38,098,270	37,457,442 (98.32%)	5,447,888,065	98.91	95.69
BDR-1b	36,410,500	35,717,738 (98.10%)	5,168,071,209	98.84	95.49
BDR-1c	43,882,736	43,128,824 (98.28%)	6,241,238,261	98.90	95.64

Note: BL, BR, BDL and BDR refer to leaf, root, drought leaf, drought root of *B*. *chinense* respectively. Raw reads number: The number of raw sequence data. Clean reads number: The filtered sequencing data. Clean bases: The total number of bases in clean data. Q20 and Q30 represent the percentage of bases with mass values greater than 20 or 30, respectively.

**Fig 2:**
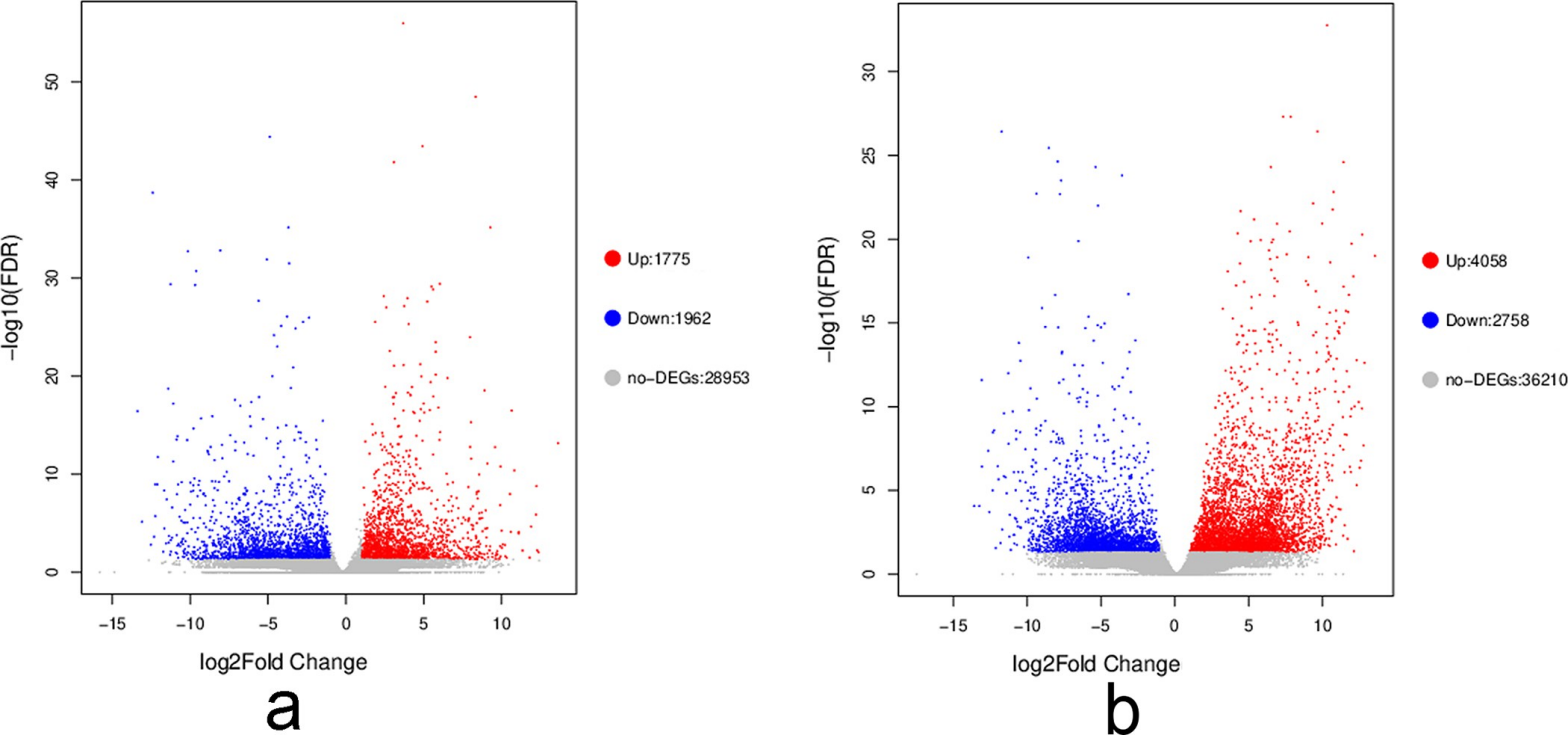
Volcano plot of differentially expressed genes of *Bupleurum chinense*. a. Leaves. b. Roots. FDR: False discovery rate.

To explore the response of *B*. *chinense* to drought stress, we identified DEGs using the criteria of FDR < 0.01 and |log2FC| > 1. In the leaves subjected to drought treatment, a total of 3737 DEGs were identified, with 1962 (52.50%) up-regulated and 1775 (47.50%) down-regulated (Fig 2). Similarly, in the drought-treated roots, a total of 6816 DEGs were identified, with 2758 (40.46%) up-regulated and 4058 (59.54%) down-regulated. In these DEGs, 382 up-regulated DEGs and 495 down-regulated DEGs were collectively involved in the response of *B*. *chinense* leaves and roots to drought stress. Additionally, 1383 genes were specifically up-regulated in leaves, while 3639 genes were up-regulated in roots. These results indicate a significant increase in the number of DEGs in roots under drought stress.

**Fig 3:**
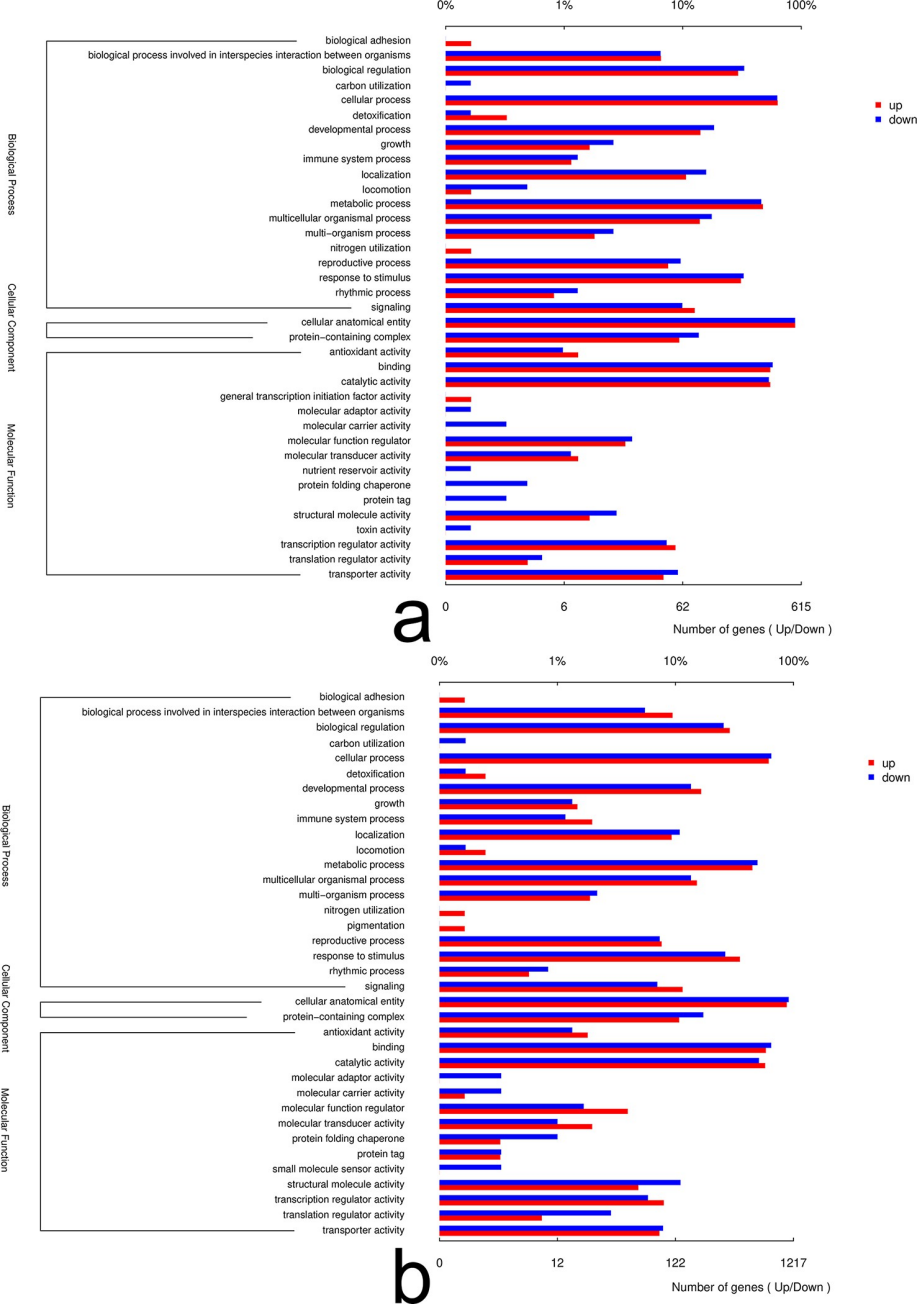
GO functional annotation histogram of differentially expressed genes of *Bupleurum chinense* after drought treatment. a. leaves. b. roots. The abscissa at the bottom indicates the number of genes annotated to a certain GO term, and the top indicates the ratio of the number of genes annotated to a certain GO term to the total number of genes annotated by GO database. The ordinate represents the primary and secondary classifications of GO.

To further investigate the functions of DEGs in *B*. *chinense* seedlings, we undertook a Gene Ontology (GO) function enrichment analysis. This analysis disclosed significant enrichment of DEGs across 37 GO terms in the leaves and 36 in the roots (Fig 3). Among the primary GO categories—cellular component (CC), biological process (BP), and molecular function (MF), BP was predominant in both roots and leaves, followed by CC and MF.

In terms of biological processes, we found that the DEGs in both roots and leaves, following drought treatment, were mainly involved in biological regulation, cellular and metabolic processes, and stimulus response. As for molecular function, the DEGs showed enrichment in subterms like signal binding and catalytic activity. For cellular components, the enriched subterms predominantly pertained to cellular anatomical structures. Notably, certain GO terms were exclusively enriched in either leaves or roots. The leaves demonstrated specific enrichment in terms like general transcription initiation factor activity, nutrient reserve activity, and toxin activity. In contrast, the roots showed unique enrichment in terms like pigmentation and small-molecule sensor activity. It’s also significant to highlight that the count of down-regulated genes in the roots was double that in the leaves.

**Fig 4:**
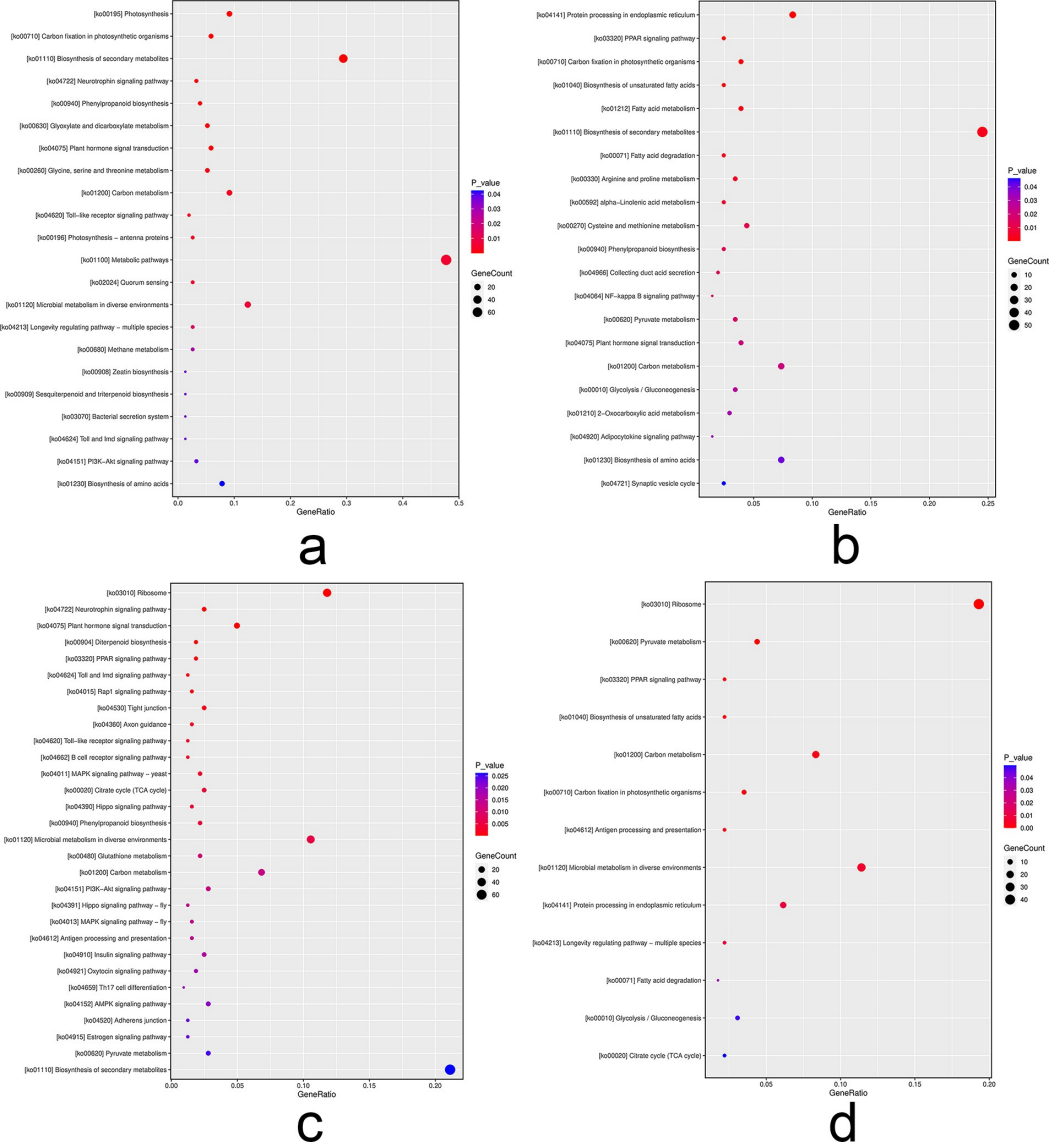
Bubble diagram of KEGG enrichment pathway of significant differentially expressed genes (DEGs) in *Bupleurum chinense* seedlings after drought treatment. a. Up-regulated DEGs in leaves. b. Down-regulated DEGs in leaves. c. Up-regulated DEGs in roots. d. Down-regulated DEGs in roots. The abscissa represents the enrichment rate, and the formula is as follows, Enrich factor = GeneRatio/BgRatio. The ordinate represents the pathway type of the KEGG. The color indicates that the significance of enrichment, that is, the *P* value. The darker the color is, the more significant the enrichment is. The right color gradient represents the size of *P* value.

In our quest to pinpoint differentially expressed genes (DEGs) and their involvement in signal transduction and biochemical metabolic pathways, we performed a KEGG enrichment analysis. The pivotal role of this analysis laid in elucidating the ramifications of drought stress. Specifically, we identified 335 DEGs in the leaves and 470 DEGs in the roots, distributed across 222 and 253 metabolic pathways, respectively (Fig 4). Notably, carbon metabolism and plant hormone signal transduction pathways were commonly affected in both leaves and roots. Furthermore, we employed hierarchical clustering of DEGs, effectively visualized by integrating FPKM values with color coding (S2 Fig). This innovative approach underscored the prominence of DEGs in these pathways. Our results unmistakably demonstrate that drought stress exerts a substantial influence on metabolic and signal transduction pathways in both the roots and leaves of *B*. *chinense*.

Post-drought stress analysis revealed specific pathways that were most affected. In leaves, the pathways with the highest enrichment included carbon fixation in photosynthetic organisms, biosynthesis of secondary metabolites, photosynthesis, and phenylpropanoid biosynthesis. Contrastingly, in roots, the significantly enriched pathways post-stress encompassed the citrate cycle (TCA cycle), diterpenoid biosynthesis, microbial metabolism in diverse environments, plant hormone signal transduction, biosynthesis of unsaturated fatty acids, and pyruvate metabolism.

### qPCR validation of DEGs

**Fig 5:**
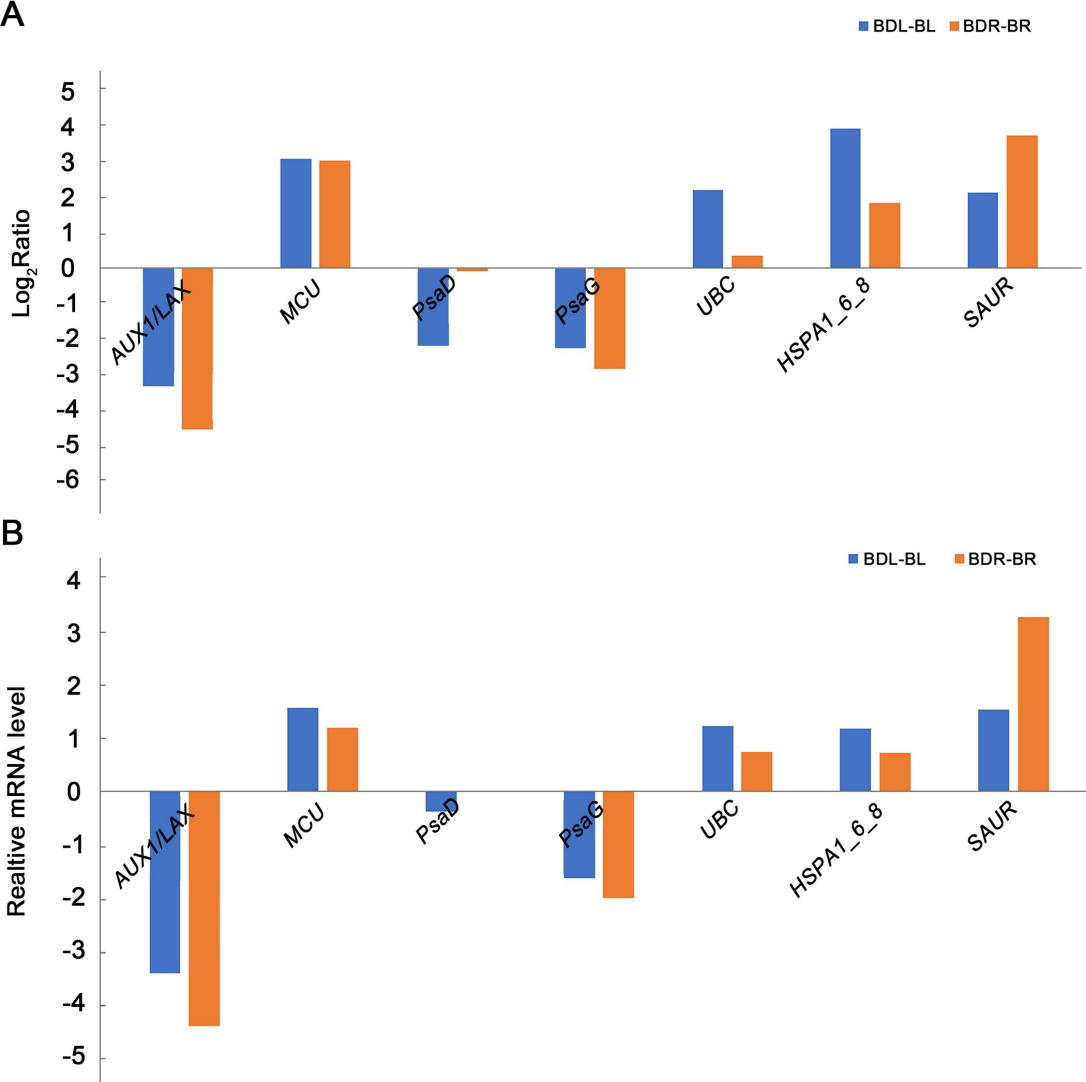
Transcriptome and real-time fluorescence quantitative PCR analysis of 7 differentially expressed genes (DEGs) in *Bupleurum chinense* seedlings. a. seven DEGs transcriptomes. The ordinate represents the logarithmic value of the expression multiples of the experimental group and the control group with a base of 2. b. Seven DEGs based on qPCR. Abbreviations: BDL-BL: Comparison of leaves after and before drought stress; BDR-BR: Comparison of roots after and before drought stress; *AUX1/LAX*—auxin influx carrier; *MCU*—calcium uniporter protein; *PsaD*—photosystem I subunit II; *PsaG*—photosystem I subunit V; *UBC*—ubiquitin C; *HSPA1_6_8*—heat shock 70kDa protein 1/6/8; *SAUR*—SAUR family protein.

To validate the expression patterns in the roots and leaves of *B*. *chinense* seeding, a total of seven DEGs with significant differential expression were selected for qPCR validation. The results from both the transcriptome analysis (Fig 5A) and qPCR experiments (Fig 5B) exhibited similar expression patterns, confirming the reliability of the transcriptome results in identifying DEGs in this study.

### Metabolomics anaylsis

**Fig 6:**
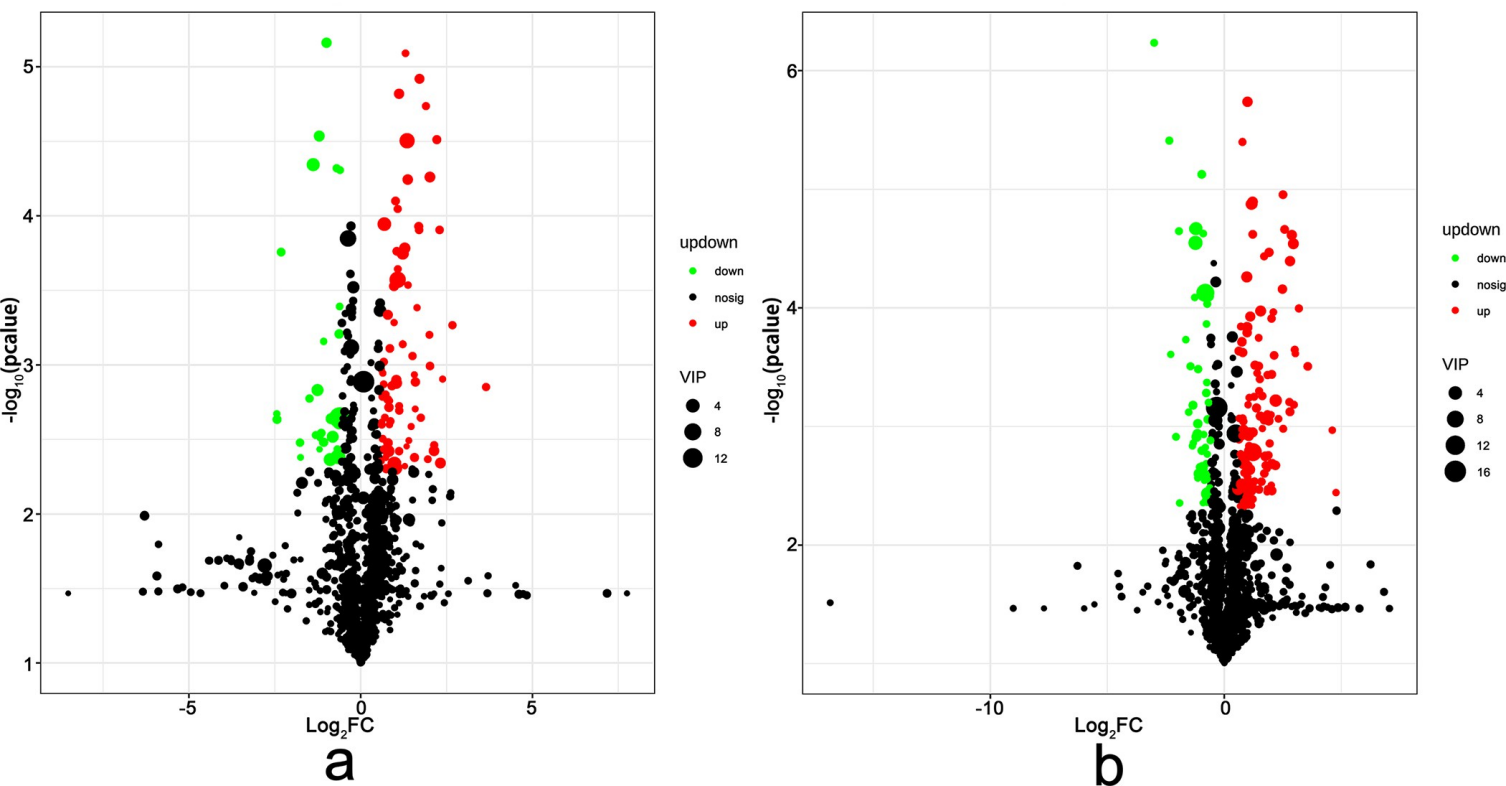
Volcano plot of differentially accumulated metabolites (DAMs) of *Bupleurum chinense* under drought stress. a. Leaves. b. Roots. Red represents up-regulated expression, green represents down-regulated expression, and black represents no difference in metabolites. The abscissa is the Fold change of DAMs between samples, and the ordinate is the statistical test value of metabolite difference, *i*.*e*., P value. The higher the P value is, the more significant the expression difference is. The X and Y values are logarithmized.

**Fig 7:**
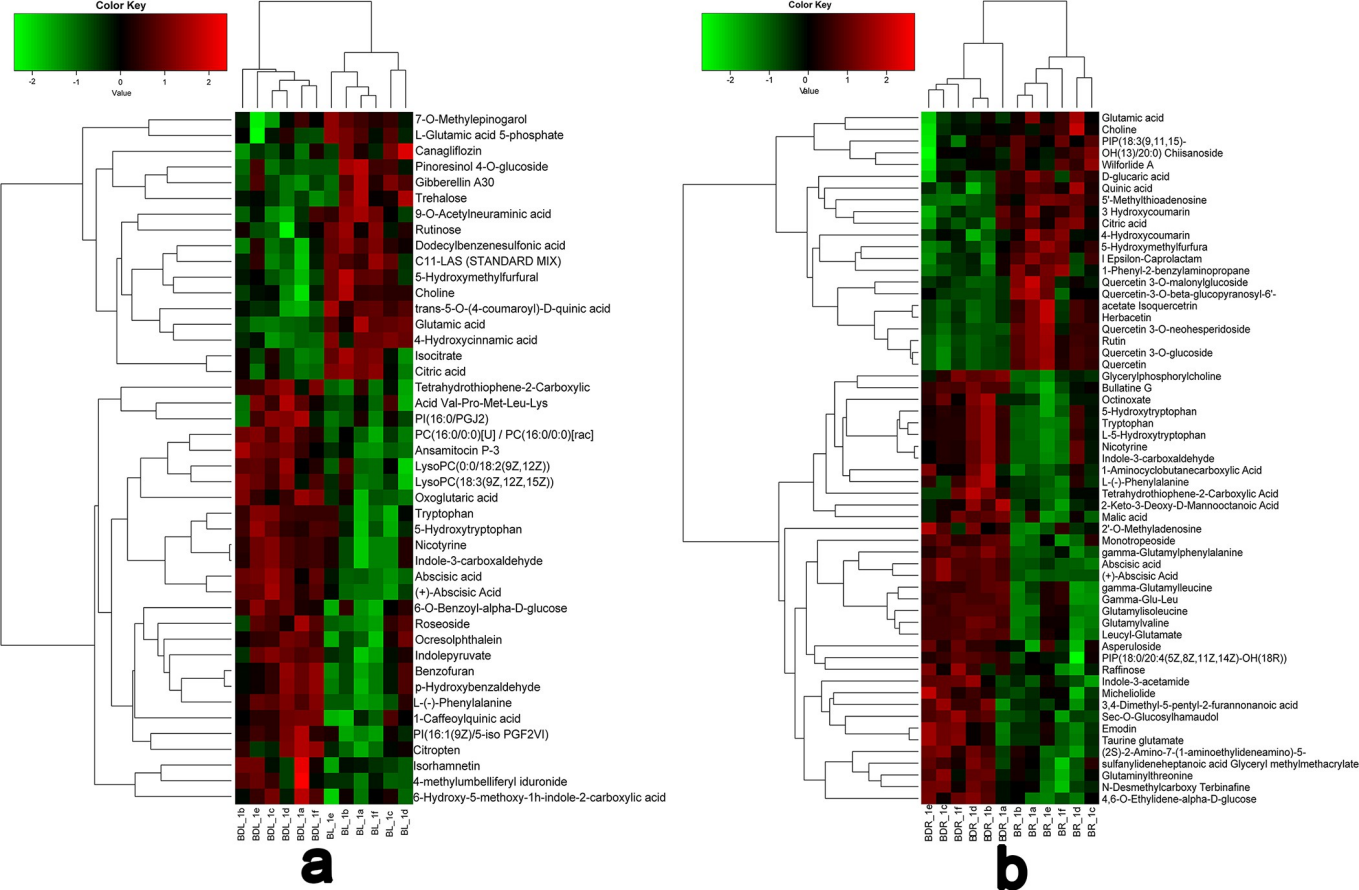
Hierarchical Clustering of differentially accumulated metabolites obtained from *Bupleurum chinense* under drought stress. a. Leaves. b. Roots. Red represents up-regulated expression, green represents down-regulated expression.

In this study, we performed a metabolomics analysis to explore the metabolites associated with drought stress. To accurately screen labeled metabolites and visually depict the sample relationships and differences in metabolite expression patterns, we employed stratification and clustering based on the expression levels of DAMs (Figs 6 and 7). A total of 44 DAMs in the leaves and 59 DAMs were identified in the roots of *B*. *chinense* seedlings. Among these DAMs, we observed 27 up-regulated metabolites and 17 down-regulated metabolites in the leaves, while in the roots, we found 37 up-regulated metabolites and 22 down-regulated metabolites. Interestingly, we identified twelve metabolites that were present in both the roots and leaves. Specifically, L-Glutamate acid, choline, citrate acid, and 5-hydroxymethylfurfural were down-regulated, whereas 5-hydroxytryptophan, tryptophan, nicotinic, indole-3-formaldehyde, L-(-)-phenylalanine, tetrahydrothiophene 2-carboxylic acid, abscisic acid, and (+)-abscisic acid were up-regulated.

To evaluate the variations in root and leaf samples of *B*. *chinense* seedlings, principal component analysis (PCA) was conducted using R (S3 Fig). The analysis revealed that accumulative variance contribution rate was 41.80% for leaf samples and 41.30% for roots samples, indicating significant differences between the two groups. Additionally, cluster analysis further confirmed the dissimilarities between the leaf and root samples, thereby demonstrating the effectiveness of the PCA models in capturing these differences.

Cluster analysis revealed significant differences between the leaf and root groups. Hence, these two PCA models effectively elucidate the dissimilarities between the two sample sets. Furthermore, we applied orthogonal partial least squares discriminant analysis (OPLS-DA) to both leaves and roots (S4 Fig). The validity of the models was confirmed through 200 permutation tests. Using the OPLS-DA model and t-test, we identified metabolic biomarker that exhibited significant differences between the groups based on a VIP score exceeding 1 and a P value below 0.05.

**Fig 8:**
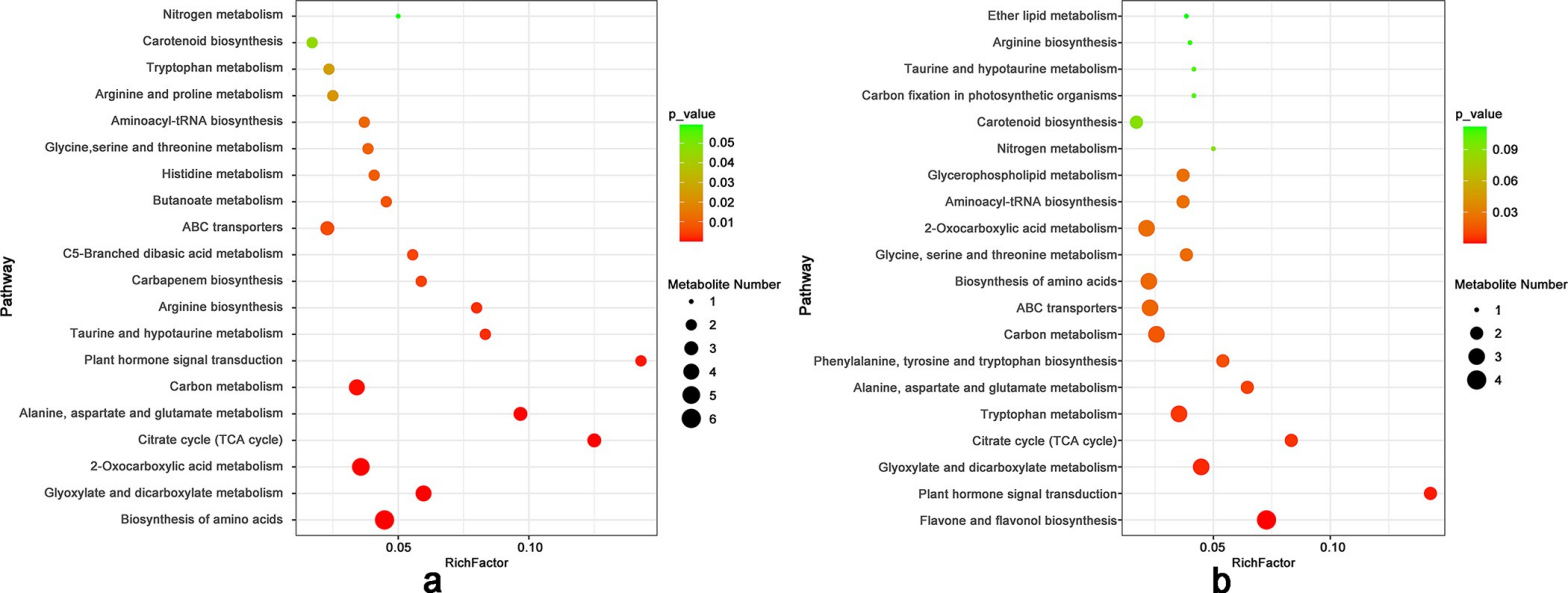
Bubble diagram of KEGG enrichment pathway of significant differentially accumulated metabolites in *Bupleurum chinense* seedlings after drought treatment. a. Leaves. b. Roots. The abscissa represents the enrichment rate, and the formula is as follows, Enrichment Ratio = Sample Number/Background Number. The ordinate represents the pathway type of the KEGG. The color indicates that the significance of enrichment, that is, the *p*-value. The darker the color is, the more significant the enrichment is. The right color gradient represents the size of P value.

The KEGG enrichment analysis of DAMs in leaves and roots revealed unique and common metabolic pathways under drought stress (Fig 8). Leaves predominantly exhibit pathways like amino acid biosynthesis, glyoxylate and dicarboxylate metabolism, 2-oxuronic acid metabolism, the citrate (TCA) cycle, and metabolism involving alanine, aspartate, glutamate, and carbon. Conversely, roots display significant activity in flavone and flavonol biosynthesis, plant hormone signal transduction, glyoxylate and dicarboxylate metabolism, the citrate cycle, tryptophan metabolism, and alanine, aspartate, and glutamate metabolism. Common metabolic pathways in both leaves and roots include amino acid biosynthesis, glyoxylate and dicarboxylate metabolism, the citrate cycle, metabolism of alanine, aspartate, glutamate, carbon, plant hormone signal transduction, and ABC transporters.

### Integrated analysis of metabolic and transcriptomic data

**Fig 9:**
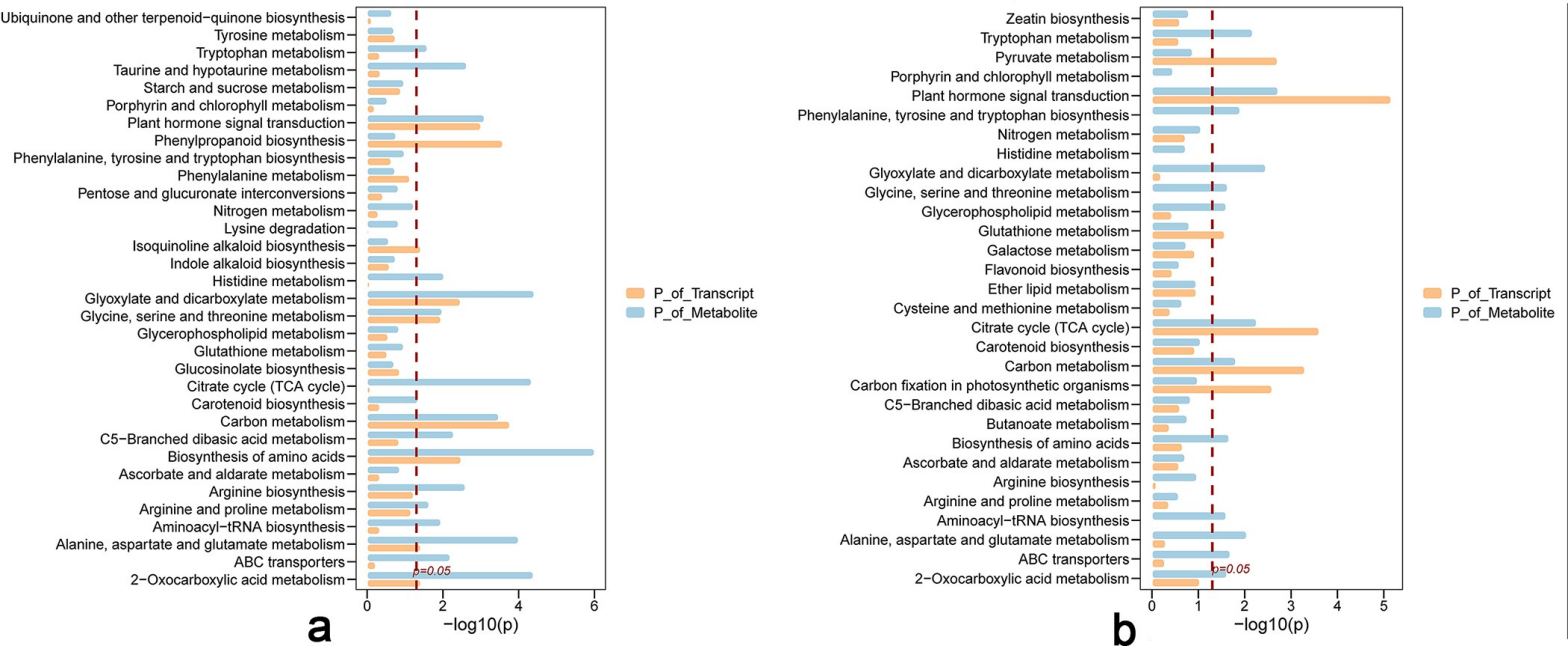
Combined analysis of differentially expressed genes and differentially accumulated metabolites. a. Leaves. b. Roots. The left side of the red line represents genes and metabolic pathways selected at *p* > 0.05 and the right side of the red line represents genes and metabolic pathways selected at *p* < 0.05.

**Fig 10:**
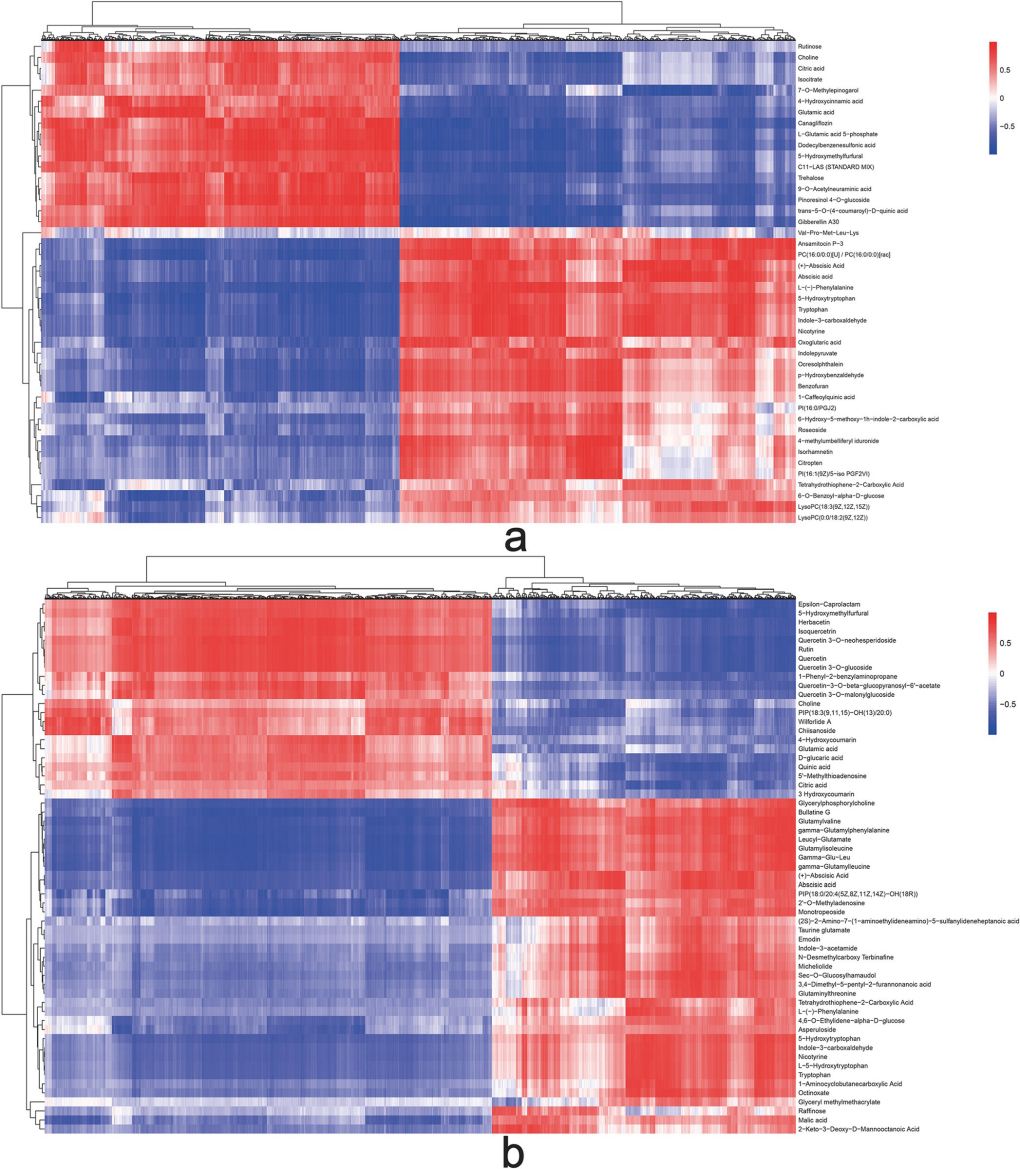
Heatmap of the correlation between differentially expressed genes and differentially accumulated metabolites from *Bupleurum chinense* under drought stress. a. Leaves. b. Roots. Pearson correlation is used to calculate the correlation coefficient (r) for the correlation between metabolites. The correlation degree between two variables is expressed by the correlation coefficient (r). it is a positively correlated, when r is higher than zero, and negatively correlated when r is less than zero.

**Fig 11:**
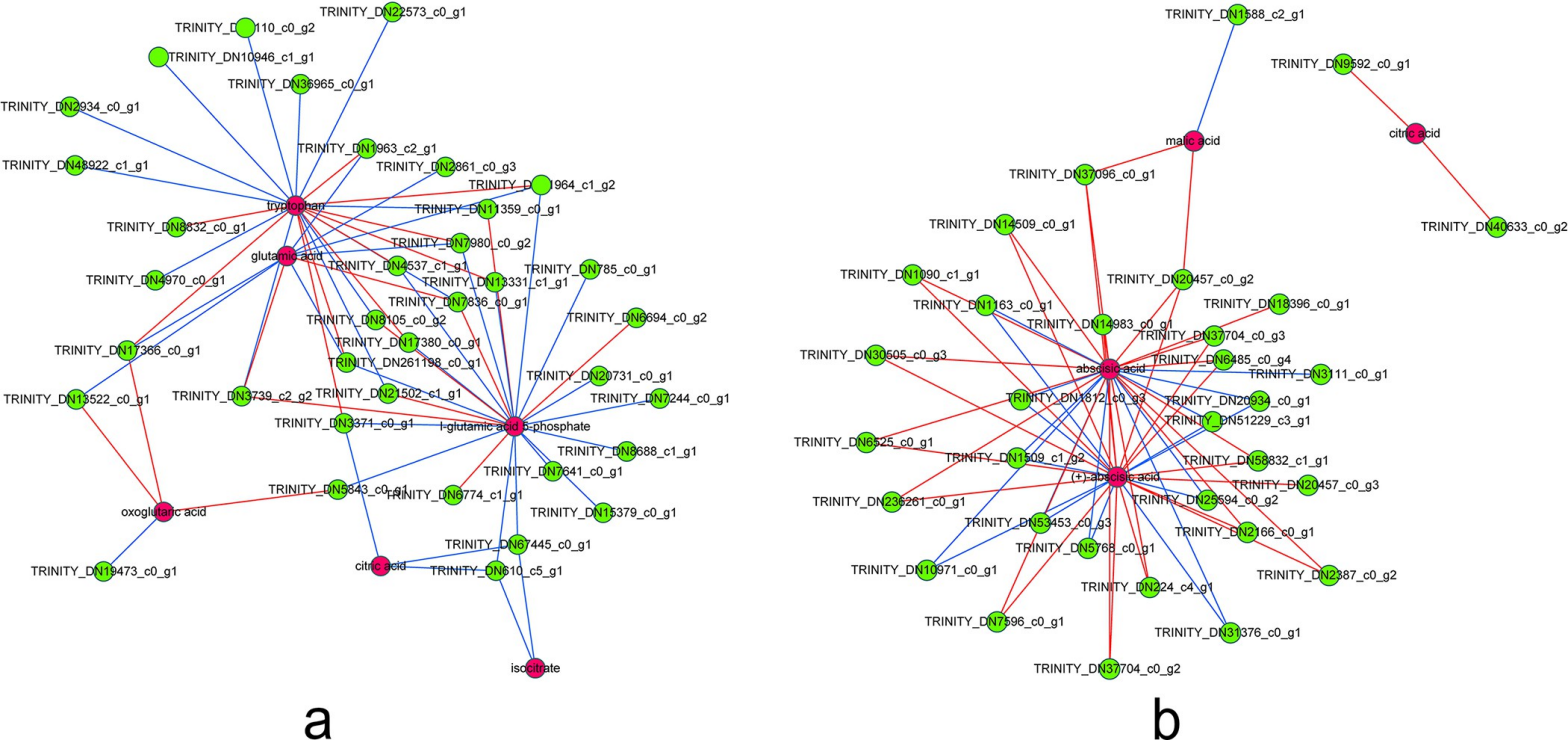
Correlation network diagram of differentially expressed genes and differentially accumulated metabolites. a. Leaves. b. Roots. Green nodes indicate genes and red nodes indicate metabolites. Blue lines indicate gene down-regulation and red line indicate gene up-regulation.

To further explore the regulatory mechanisms in *B*. *chinense* seedlings under drought stress, we conducted a comprehensive analysis of transcriptional and metabolic data. The results demonstrate significant correlations between numerous differential genes and metabolites (Fig 9). Our comparative analysis of transcriptome and metabolome pathways (Fig 10) revealed that many significant DAMs and DEGs were enriched in diverse pathways. Noticeably, the most enriched pathway in leaves was amino acids biosynthesis, with 81 DEGs and 8 DAMs, followed by glyoxylate and dicarboxylic acid metabolism with 38 DEGs and 4 DAMs. In contrast, roots exhibited the highest enrichment in plant hormone signal transduction, with 72 DEGs and 2 DAMs, and the citrate cycle (TCA cycle) with 62 DEGs and 2 DAMs. A correlation network diagram (Fig 11) further elucidates the relationship between DAMs and DEGs enriched in the same KEGG pathways in both leaves and roots.

### Regulation of carbon and nitrogen metabolism

**Fig 12:**
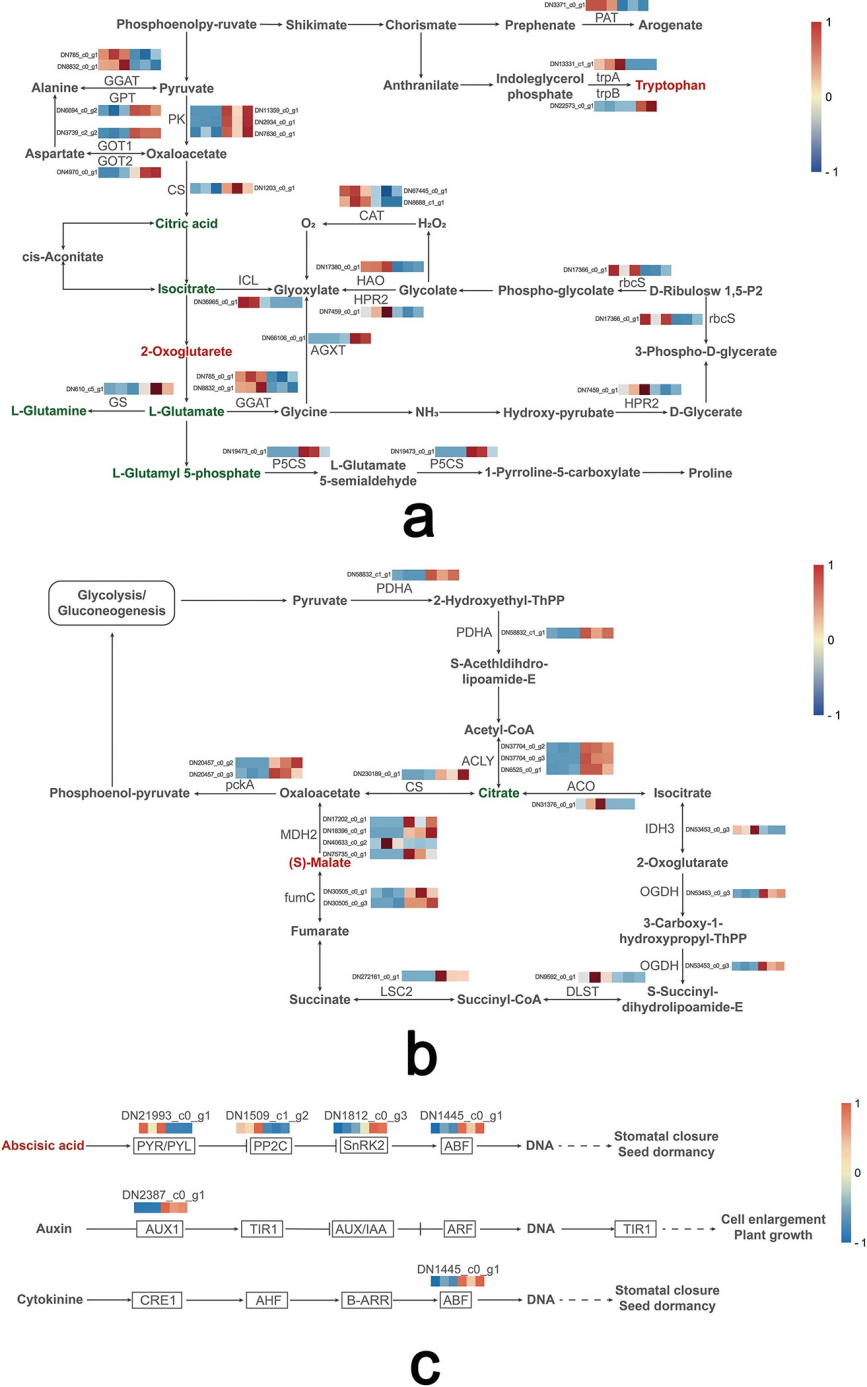
Transcriptomic and metabolic shifts of *Bupleurum chinense* seedlings under drought stress. Upregulation is indicated in red and downregulation is indicated in blue (genes) or green (metabolites). a. Carbon and nitrogen metabolism in *B*. *chinense* seedlings. b. TCA cycle in *B*. *chinense* seedlings. c. Plant hormone signal transduction pathway in *B*. *chinense* seedlings.

The integrated of transcriptome and metabolome analyses revealed that amino acid biosynthesis and glyoxylate synthesis and dicarboxylate metabolism pathway were the two most significantly enriched pathways in leaves of *B*. *chinense* under drought stress. Many genes and compounds associated with these metabolic pathways played a role in maintaining the balance of carbon and nitrogen metabolism in plants. Therefore, our analysis was concentrated on the key DEGs and DAMs within these pathways (Fig 12A). The result showed that five DAMs, namely citric acid, isocitrate, L-Glutamate, L-Glutamine and L-glutamic acid 5-phosphate were down-regulated. In contrast, two DAMs, tryptophan and 2-Oxoglutarete, were up-regulated in leaves after drought stress. Transcriptome analysis revealed significant alterations in gene expression levels: GS (DN610_c5_g1), GOT1 (DN3739_c2_g2) and GOT2 (DN4970_c0_g1) showed increased expression, while the expression of GGAT (DN785_c0_g1 and DN8832_c0_g1) experienced a decrease under drought stress compared to the control group. Additionally, ICL (DN36965_c0_g1) was down-regulated, whereas GPT (DN6694_c0_g2) were respectively up-regulated. Notably, the expression of trpA (DN13331_c1_g1) was down-regulated, whereas trpB (DN22573_c0_g1) was up-regulated, indicating a coordinated regulation in the production of tryptophan.

### Regulation of ABA signaling and TCA cycle

The comparative analysis of root data from *B*. *chinense* seedlings indicated a notable enrichment in the plant hormone signal transduction pathway (ko04075) under drought stress (Fig 12C). Within this pathway, we identified 5 DEGs associated with phytohormone signaling. Among these, the *PYR/PYL* gene (DN21993_c0_g1) displayed significant up-regulation, whereas the expression of *PP2C* gene (DN1509_c1_g2) was down-regulated. Furthermore, the expression of *SnRK2* gene (DN1812_c0_g3) was up-regulated, while the *ABF* gene (DN1445_c0_g1) was down-regulated. Additionally, the expression of the *AUX1* gene (DN2387_c0_g1) was found to be up-regulated. Metabolomics analysis revealed a significant up-regulation of abscisic acid.

Another pathway that exhibited a notably shift in response to drought stress is the citrate cycle (Fig 12B). The result revealed that nine DEGs associated with the citrate cycle were significantly up-regulated, whereas four DEGs were markedly down-regulated. Specifically, the *MDH2* gene, with its variants DN17202_c0_g1, DN18396_c0_g1, DN40633_c0_g2, and DN75735_c0_g1, along with fumC (DN30505_c0_g1, DN30505_c0_g3), showed an up-regulation. In contrast, the expressions of *CS* gene (DN230189_c0_g1) and *ACLY* (DN6525_c0_g1) were found to be down-regulated, Meanwhile, the *ACO* gene (DN31376_c0_g1) exhibited an increase in expression. Complementary to these transcriptomic insights, metabolomics analysis identified significant variations in the levels of key metabolites within the citric acid cycle pathway. Specifically, there was a decrease in citric acid levels, whereas malic acid levels were found to be up-regulated.

## Discussion

This research provides a detailed multi-omics examination of the molecular mechanisms governing the response of *B*. *chinense* seedlings to drought stress. The integration of physiological, transcriptomic, and metabolomic analyses has provided a deeper understanding of the adaptive strategies employed by this medicinal plant in response to drought [40,41].

Key in our findings is the distinct behavior of antioxidative enzymes like POD, SOD, and CAT in different parts of plants, indicating a spatially varied oxidative stress response. The roots, being the primary contact with soil, act as the initial line of defense against ROS [42], with leaves reacting subsequently. This sequential response is evident in the increased CAT activity in leaves, suggesting its pivotal role in countering oxidative damage, and the relative stability of SOD activity in roots, underscoring its significance in the subterranean sections of plants.

Transcriptome analysis revealed a notable increase in DEGs in the roots compared to the leaves, reflecting a more pronounced genomic response to drought stress in roots. This finding is consistent with the roots being the initial point of contact with the drying soil conditions. The variation in GO terms between leaves and roots highlights the specific responses of these organs to drought, showing differences in biological processes, molecular functions, and cellular components. Additionally, KEGG pathway analysis revealed the involvement of numerous metabolic pathways, particularly carbon metabolism and plant hormone signal transduction, which are crucial for the plant’s adaptation to drought [43,44].

The maintenance of carbon and nitrogen assimilation is crucial for drought tolerance, with leaves being the site where these processes interact [45]. Drought stress up-regulates 2-oxoglutarate expression in *B*. *chinense* leaves, a key component of carbon and nitrogen metabolism [46]. Further, transcriptome analysis showed increased expression of genes associated with amino acid biosynthesis (e.g., GS gene, DN610_c5_g1) and decreased expression of key enzymes in plant nitrogen assimilation (e.g., GGAT gene, DN8832_c0_g1 and DN785_c0_g1) under drought stress. These adjustments likely play a critical role in drought response of *B*. *chinense* by maintaining photosynthetic efficiency and nutrient assimilation [47,48].

Notably, drought stress decreased isocitric acid level in leaves of *B*. *chinense* seedlings and down-regulated the expression of ICL (DN36965_c0_g1), a key enzyme in glyoxylic acid cycle [49]. The primary function of ICL is to catalyze the breakdown of isocitric acid into glyoxylate and succinate, influencing the flow of carbon sources [50]. This suggests that drought stress may inhibit the TCA cycle, affecting the metabolism of glyoxylic and dicarboxylic acid in the *B*. *chinense* seedlings.

Our study highlights the significance of ABA signaling in drought response, especially in roots. The accumulation of abscisic acid (ABA) in *B*. *chinense* seedling roots underscores its regulatory role during drought. The ABA receptor protein PYL (DN21993_c0_g1) plays a critical part in recognizing ABA signals and initiating signal transduction [51]. Transcriptomics data revealed an up-regulation of PYL and SnRK2, and a down-regulation of PP2C and ABF following drought-induced ABA production, consistent with previous research [52] ABA is instrumental in modulating the root/shoot ratio, promoting root growth, and aiding in soluble sugar accumulation and distribution under osmotic stress, which explains the higher root-shoot ratio in *B*. *chinense* seedlings under drought stress[53,54] (S5 Fig).

The increased expression of MDH2 (DN17202_c0_g1) and fumC (DN30505_c0_g1 and DN30505_c0_g3) in the TCA cycle of *B*. *chinense* seedlings under drought stress suggests a metabolic adjustment to reduce oxidative stress and enhance stress resistance [55,56]. Overexpression of MDH2 aids in regulating organic acid transport and solute potential, thereby diminishing ROS production and enhancing stress resistance [57,58]. FumC and MDH collaborate to regulate malate levels, with a negative correlation between malic acid. Accumulation of malic acid in the roots may regulate stomatal closure and improve stress tolerance in *B*. *chinense* seedlings under drought conditions.

In summary, this study highlights the intricate and multi-faceted molecular response of *B*. *chinense* seedlings to drought stress. The findings contribute to a more comprehensive understanding of drought tolerance mechanisms in medicinal plants, providing potential insights for future agricultural and biotechnological applications aimed at enhancing drought resistance in crops.

## Conclusions

In conclusion, this study comprehensively investigated the impacts of drought stress on *B*. *chinense* seedlings through physiological, biochemical, metabolomics, and transcriptomics analyses. The results revealed significant changes in the expression of gene and metabolite levels in response to drought stress in both the leaves and roots of *B*. *chinense*. The integrated analysis of transcriptome and metabolome data highlighted the importance of carbon-nitrogen metabolism and phytohormone signaling in the response of *B*. *chinense* seedlings to drought stress. Notably, abscisic acids and organic acids involved in the TCA cycle and amino acid biosynthesis were identified as key factors contributing to stress resistance in *B*. *chinense* seedlings. This study enhanced the understanding of the molecular mechanisms underlying drought resistance in *B*. *chinense*, provides a basis for the identification of drought-tolerant genes, and serves as a valuable resource for the selection of drought-resistant varieties.

## Supporting information

S1 Fig PCA analysis based on the expression of unigenes. The X and Y axes represent the new data set of the corresponding principal components obtained after dimensionality reduction of the sample expression, which is used to indicate the degree of dispersion between samples. The value in the axis label represents the percentage of the overall variance explained by the corresponding principal components. The same color represents the same group of samples, and different shapes represent different groups of samples.
(TIF)

S2 Fig Heat map of expression patterns of differentially expressed genes (DEGs). a. BDL vs BL. b. BDR vs BR. Each column in the figure represents a sample, and each row represents a gene. The color in the figure represents the expression level of the gene in the group of samples (log10 FPKM). Red and green represent trends in gene expression levels. Please refer to the numerical label under the color bar at the upper right.
(TIF)

S3 Fig Score plot of PCA model. a. Leaves. b. Roots. The abscissa represents the first principal component in the original data, and the ordinate represents the second principal component in the original data. The samples are clustered together, indicating small differences between the samples. On the contrary, the samples presented obvious differences when they were not in the same location.
(TIF)

S4 Fig Score plot of OPLS-DA model. a. Leaves. b. Permutation testing of model. c. Roots. d. Permutation testing of model. Permutation test is a method to evaluate the accuracy of OPLS-DA model. All blue Q2-values to the left are lower than the original points to the right, or the blue regression line of the Q2-points intersects the vertical axis (on the left) at, or below zero, indicating that the model fitting effect is good.
(TIF)

S5 Fig . Comparison of seedling morphology between control group and drought treatment group. a. Control group. b. Treatment group.
(TIF)

S1 Table Samples of Bupleurum chinense in transcriptomics and metabolomics. (DOCX)

S2 Table S2 Table RNA quality test results of 12 Bupleurum chinense samples. (DOCX)
