# Supplementary figures and images for: Integrated physiological, transcriptomics and metabolomics analysis revealed the molecular mechanism of Bupleurum chinense seedlings to drought stress
Source: PLoS One. 2024 Jun 6;19(6):e0304503. doi: 10.1371/journal.pone.0304503 (PMC11156411; doi:10.1371/journal.pone.0304503)

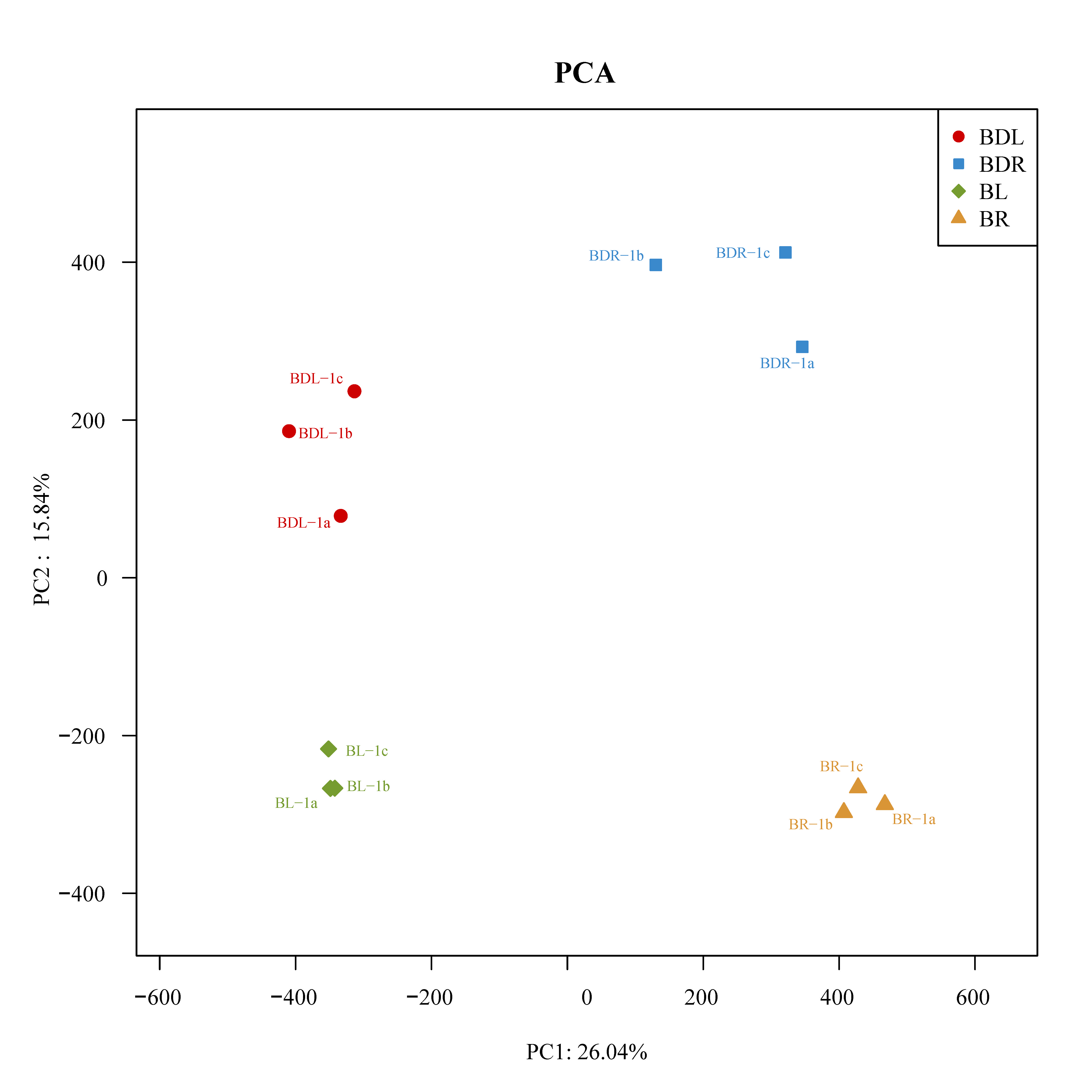

Supplement: S1 Fig — The X and Y axes represent the new data set of the corresponding principal components obtained after dimensionality reduction of the sample expression, which is used to indicate the degree of dispersion between samples. The value in the axis label represents the percentage of the overall variance explained by the corresponding principal components. The same color represents the same group of samples, and different shapes represent different groups of samples. (TIF) [file pone.0304503.s001.tif]

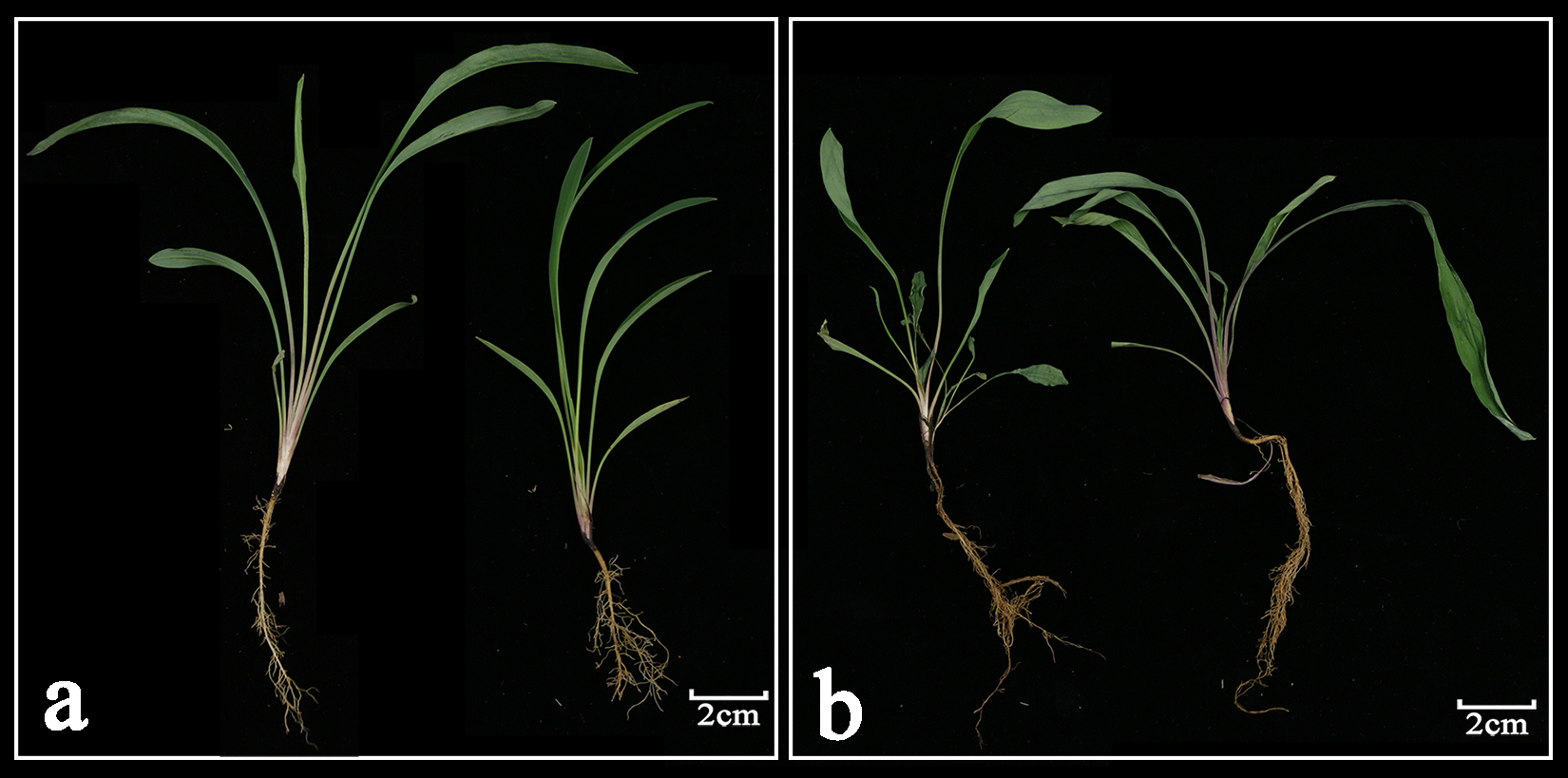

Supplement: S5 Fig — a. Control group. b. Treatment group. (TIF) [file pone.0304503.s005.tif]
