# Supplementary material for: Integrated physiological, transcriptomics and metabolomics analysis revealed the molecular mechanism of Bupleurum chinense seedlings to drought stress
Source: PLoS One. 2024 Jun 6;19(6):e0304503. doi: 10.1371/journal.pone.0304503 (PMC11156411; doi:10.1371/journal.pone.0304503)
Supplement: S1 Table — (DOCX) [file pone.0304503.s006.docx]

Table S1 Samples of *Bupleurum chinense* in transcriptomics and metabolomics

| Omics | Leaf | | Root | |
| --- | --- | --- | --- | --- |
|  | Control group | Experimental group | Control group | Experimental group |
| Transcriptomics  （n=3） | BL-1a | BDL-1a | BR-1a | BDR-1a |
|  | BL-1b | BDL-1b | BR-1b | BDR-1b |
|  | BL-1c | BDL-1c | BR-1c | BDR-1c |
| Metabolomics  （n=6） | BL-1a | BDL-1a | BR-1a | BDR-1a |
|  | BL-1b | BDL-1b | BR-1b | BDR-1b |
|  | BL-1c | BDL-1c | BR-1c | BDR-1c |
|  | BL-1d | BDL-1d | BR-1d | BDR-1d |
|  | BL-1e | BDL-1e | BR-1e | BDR-1e |
|  | BL-1f | BDL-1f | BR-1f | BDR-1f |

Note: BL: The leaf of *B. chinense*. BR: The root of *B. chinense*. BDL: The drought leaf of *B. chinense*. BDR: The drought root of *B. chinense.*
