# Supplementary material for: Integrated physiological, transcriptomics and metabolomics analysis revealed the molecular mechanism of Bupleurum chinense seedlings to drought stress
Source: PLoS One. 2024 Jun 6;19(6):e0304503. doi: 10.1371/journal.pone.0304503 (PMC11156411; doi:10.1371/journal.pone.0304503)
Supplement: S2 Table — (DOCX) [file pone.0304503.s007.docx]

Table S2 RNA quality test results of 12 *Bupleurum chinense* samples

| Samples | Concentration (ng/μL) | OD_260/280_ | OD_260/230_ |
| --- | --- | --- | --- |
| BL-1a | 771.1 | 2.18 | 2.07 |
| BL-1b | 734.1 | 2.17 | 2.32 |
| BL-1c | 650.6 | 2.14 | 2.24 |
| BR-1a | 194.4 | 2.15 | 1.82 |
| BR-1b | 319.2 | 2.12 | 2.24 |
| BR-1c | 412.3 | 2.09 | 2.03 |
| BDL-1a | 940.2 | 2.17 | 2.09 |
| BDL-1b | 1138.6 | 2.14 | 2.36 |
| BDL-1c | 801.5 | 2.17 | 2.27 |
| BDR-1a | 312.2 | 2.08 | 1.96 |
| BDR-1b | 275.5 | 2.07 | 1.92 |
| BDR-1c | 313.0 | 1.97 | 1. 94 |

Note: BL: The leaf of *B. chinense* BR: The root *B. chinense.* BDL: The drought leaf *B. chinense*. BDR: The drought root *B. chinense*.
